# Reference state and benchmark concepts for better biodiversity conservation in contemporary ecosystems

**DOI:** 10.1111/gcb.15383

**Published:** 2020-10-23

**Authors:** Megan J. McNellie, Ian Oliver, Josh Dorrough, Simon Ferrier, Graeme Newell, Philip Gibbons

**Affiliations:** ^1^ Department of Planning, Industry and Environment Science, Economics and Insights Division Wagga Wagga NSW Australia; ^2^ Fenner School of Environment and Society The Australian National University Acton ACT Australia; ^3^ Department of Planning, Industry and Environment Science, Economics and Insights Division Gosford NSW Australia; ^4^ Department of Planning, Industry and Environment Science, Economics and Insights Division Merimbula NSW Australia; ^5^ CSIRO Land and Water Canberra ACT Australia; ^6^ Department of Environment, Land, Water and Planning Arthur Rylah Institute for Environmental Research Heidelberg Vic. Australia

**Keywords:** benchmark, composition, conceptual framework, conservation, reference state, restoration, structure, vegetation

## Abstract

Measuring the status and trends of biodiversity is critical for making informed decisions about the conservation, management or restoration of species, habitats and ecosystems. Defining the reference state against which status and change are measured is essential. Typically, reference states describe historical conditions, yet historical conditions are challenging to quantify, may be difficult to falsify, and may no longer be an attainable target in a contemporary ecosystem. We have constructed a conceptual framework to help inform thinking and discussion around the philosophical underpinnings of reference states and guide their application. We characterize currently recognized historical reference states and describe them as Pre‐Human, Indigenous Cultural, Pre‐Intensification and Hybrid‐Historical. We extend the conceptual framework to include contemporary reference states as an alternative theoretical perspective. The contemporary reference state framework is a major conceptual shift that focuses on current ecological patterns and identifies areas with higher biodiversity values relative to other locations within the same ecosystem, regardless of the disturbance history. We acknowledge that past processes play an essential role in driving contemporary patterns of diversity. The specific context for which we design the contemporary conceptual frame is underpinned by an overarching goal—to maximize biodiversity conservation and restoration outcomes in existing ecosystems. The contemporary reference state framework can account for the inherent differences in the diversity of biodiversity values (e.g. native species richness, habitat complexity) across spatial scales, communities and ecosystems. In contrast to historical reference states, contemporary references states are measurable and falsifiable. This ‘road map of reference states’ offers perspective needed to define and assess the status and trends in biodiversity and habitats. We demonstrate the contemporary reference state concept with an example from south‐eastern Australia. Our framework provides a tractable way for policy‐makers and practitioners to navigate biodiversity assessments to maximize conservation and restoration outcomes in contemporary ecosystems.

## Graphical abstract

We synthesise currently recognised historical reference states and describe them as Pre‐Human, Indigenous Cultural, Pre‐Intensification and Hybrid‐Historical. We extend the conceptual framework to include contemporary reference states as an alternative theoretical perspective. The specific context for which we design the contemporary conceptual frame is underpinned by an overarching goal—to maximise biodiversity conservation and restoration outcomes in existing ecosystems. In contrast to historical reference states, contemporary references states are measurable and falsifiable. This ‘road map of reference states’ offers perspective needed to define and assess the status and trends in biodiversity and habitats.

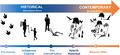

## 1 INTRODUCTION

A fundamental principle underpinning the conservation, management and restoration of species and habitats is assessing their current state against some former state or baseline. However, choosing ‘which state’ or ‘which baseline’ are questions that challenge conservation practitioners. For example, the 2019 global assessment by the Intergovernmental Science‐Policy Platform on Biodiversity and Ecosystem Services (IPBES) used multiple baselines to assess status and trends. The authors of IPBES (2019) estimated that 47% of ecosystems have declined in extent and condition when compared to ‘prehistory baseline’; habitat integrity has reduced by 30% relative to an ‘unimpacted baseline’; and that 680 vertebrate species have become extinct ‘since the year 1500’ (IPBES, 2019). Alternatively, the Living Planet Index uses a more recent, 1970 time‐stamped baseline, to assess and monitor the decline in the abundance of vertebrate populations and species (Butchart et al., 2010; Tittensor et al., 2010). These global assessments demonstrate some approaches for assessing the status and trends in biodiversity that are urgently needed to galvanize conservation efforts. However, where baselines and reference states are derived from different metrics and span timeframes from many thousands of years to fewer than 50 years, their potential for assessing biodiversity conservation and restoration outcomes in existing ecosystems can be constrained.

Because of the wide‐ranging end goals and inconsistent timeframes, the reference state concept has been criticized as being too complicated to quantify and challenging to confirm (Corlett, 2016; Hobbs et al., 2014; Hughes et al., 2017; Kopf et al., 2015; Suding, 2011). Where reference states are defined by distant, variable and non‐specific historical timeframes, these may represent impractical targets needed to guide contemporary biodiversity conservation (Suding, 2011). In addition, current environmental conditions have changed markedly from those that underpin historical reference states, as such, historical reference states may be unsuitable for contemporary and future ecosystems (Hobbs et al., 2014).

Adding to the concerns about defining historical reference states is that the benchmarks (see definitions in Box 1) used to describe historical reference states are often derived from heuristic approaches like best professional judgement or opinion (e.g. Faber‐Langendoen et al., 2012). Expert judgement is beneficial when empirical data are scarce, multiple experts can be canvassed or rapid decision support is needed to help inform policy (e.g. Gibbons et al., 2009; Sinclair et al., 2015). However, expert‐defined reference states embody several assumptions regarding disturbance patterns and processes; opinions of experts can be laden with varying historical, social, political, economic and cultural values (Burgman et al., 2011; Kahneman, 2011). For example, there is little disagreement that natural and Indigenous‐managed fires have influenced vegetation composition and structure, but there are debates and uncertainties about the extent, frequency and severity of fire regimes (Bowman et al., 2011; Denevan, 1992; Enright & Thomas, 2008; Gammage, 2011). These debates are difficult to resolve because historical, archaeological and palaeo‐ecological evidence is fragmented and can be difficult to interpret with certainty rendering expert opinion challenging to confirm.

**FIGURE 1:**
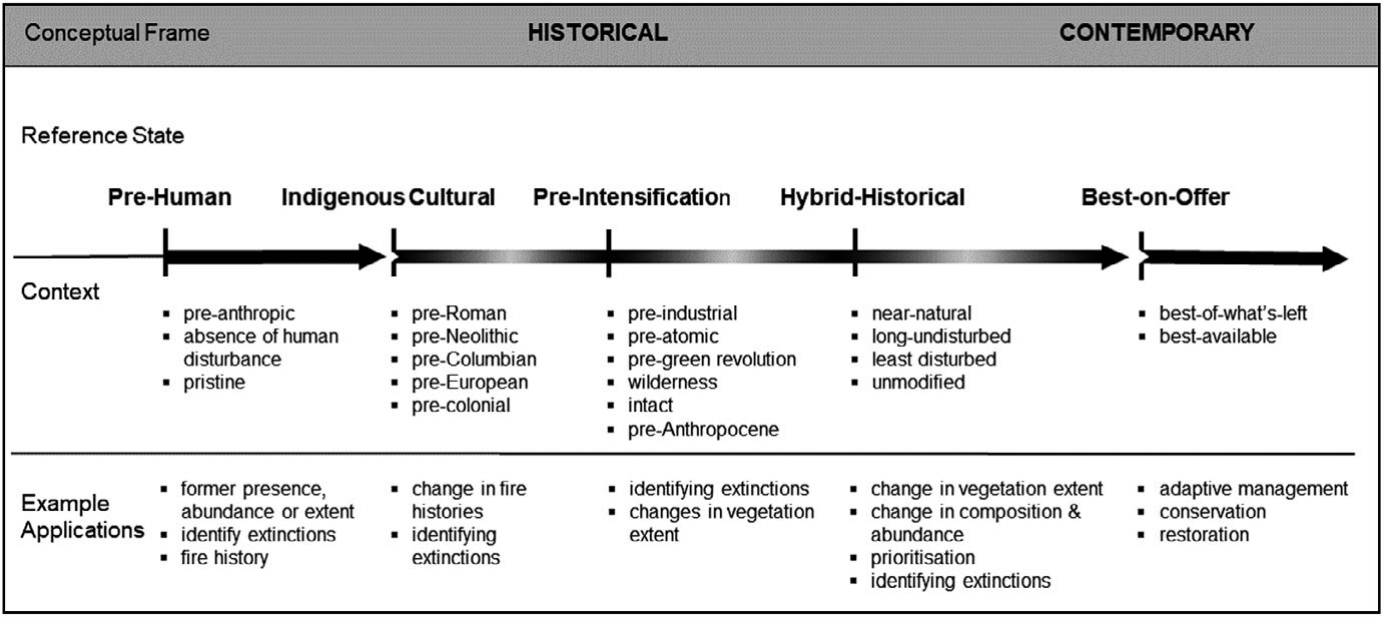
A conceptual framework for synthesizing the historical and contemporary reference states and their context and applications within the context of informing biodiversity conservation and restoration outcomes in existing ecosystems

Here we examine the historical reference state concept (see Box 1) to assess biodiversity values in the context of ecosystem conservation and management. We review the applications of historical reference states for assessing site‐scaled biodiversity values and construct a conceptual framework to understand the underpinnings of reference states and guide their application (Figure 1). We show how the definition and interpretation of reference states should be tailored to specific contexts, spatial and temporal scales. We then propose contemporary reference states as an alternative framework to inform better biodiversity conservation and management decisions in contemporary landscapes. The specific context for which we design the contemporary conceptual frame is underpinned by an overarching goal—to maximize biodiversity conservation and restoration outcomes in existing ecosystems. Finally, we illustrate an operational approach to defining site‐scaled biodiversity values for contemporary reference states and outline its benefits.

BOX 1 **Definitions of terminology used in this synthesis**

**Conceptual frame** is the *theoretical perspective* from which reference states are identified and defined. The choice of conceptual frame and the underlying assumptions are context dependent and need to be explicitly defined when assessing species, habitats or biodiversity. At the broadest level in our conceptual framework we define a dichotomy in the context and purpose for reference states, and we split them into either ***historical*** or ***contemporary*** (see Figure 1).

**Reference states**, also referred to as reference condition, are the *ecological context* for how we assess the current state of species, populations, habitats, ecosystems or biodiversity. Reference states can be specified using site‐scaled or landscape‐scaled proxies, surrogates or indicators.

**Baselines** define the *temporal dimension* across which we assess change. Timeframes for baseline reporting need to be clearly defined although the temporal specificity of the baseline can differ for different end goals.

**Benchmarks** are the *numerical context* that defines the reference state. These may be empirically or expert derived from abundance, richness or diversity of genes, populations, species, communities or their habitat, or from composite measures of biodiversity composition, structure or function (Noss, 1990).

## 2 REVIEWING THE HISTORICAL REFERENCE STATE CONCEPTUAL FRAME

Traditionally, thresholds of transformative change define historical reference states; and most often anthropogenic disturbances are the drivers of change. Here we extend the concepts proposed by Stoddard et al. (2006) and identify four distinct historical reference states as Pre‐Human, Indigenous Cultural, Pre‐Intensification and Hybrid‐Historical (Figure 1). As these reference states are conceptual, they do not necessarily have specific date stamps, nor do they have rigid or fixed temporal windows. They represent a continuum of human disturbance, and thus different types of reference states are used for conservation, management and restoration. A principal tenet of our conceptual framework is that we recognize ecosystems have transitioned through these disturbance intervals, and thus ecosystems carry a cumulative and historical legacy of change.

### 2.1 Pre‐Human reference state

Pre‐Human reference states represent the temporal baseline before human presence. Often Pre‐Human reference states are regarded as pristine and are synonymous with concepts such as or pre‐anthropic, historical or natural range of variability (Landres et al., 1999; Stoddard et al., 2006) and potential natural vegetation (Loidi & Fernández‐González, 2012). For ecosystem conservation, management and restoration of the Pre‐Human baseline can be used to evaluate changes in the extent of habitat (Zerbe, 1998) and the composition and abundance of species (Lotze & Worm, 2009; Zu Ermgassen et al., 2012), disturbance regimes and environmental conditions. Most often, Pre‐Human baselines are derived from palaeo‐ecological evidence. For example, sedimentary charcoal data infer fire variability (Froyd & Willis, 2008; Herring et al., 2018) and stable isotope and geochemical studies have helped reconstruct patterns in climate variability (Birks, 2012; McCarroll & Loader, 2004). Palaeo‐ecological information has provided a better understanding of how Pre‐Human reference states have been transformed as a result of overharvesting, overhunting, habitat transformation and mass extinctions (Burney & Flannery, 2005). Froyd and Willis (2008) suggest that Pre‐Human reference states represent long‐established patterns where ecosystems were in equilibrium with past environments. However, in most instances, inference based on Pre‐Human reference states is too sparse to be used for broader landscape‐scaled reconstructions of historical ecosystems (Seddon et al., 2014).

### 2.2 Indigenous Cultural reference state

The Indigenous Cultural reference state represents the time period between the arrival of First Nations and Indigenous people, up to colonization and is not limited to specific cultures or landmasses, but includes Roman, Columbian or European colonization. We classify pre‐colonial, pre‐Columbian, pre‐European and pre‐1610 Orbis spike as Indigenous Cultural reference states. The conceptual frame for Indigenous Cultural reference state is transferable to all locations and aims to recognize the transition in land management practices and the consequent and intensified impacts on the natural environment. Bliege Bird and Nimmo (2018) purport that Indigenous place‐based societies co‐evolved with ecosystems across vast portions of the globe. Indigenous Cultural management practices such as harvesting or burning (Anderson, 2005; Kimmerer, 2002), trapping and hunting (Rose et al., 2016) or landscape‐scaled fire management (Bowman et al., 2011; Denevan, 1992; Gammage, 2011; Nowacki & Abrams, 2008) aim to incorporate ecosystem processes such as herbivory, seed dispersal, soil turnover, predation and burning to maintain conservation, management and restoration (Bjorkman & Vellend, 2010). By restoring sophisticated and strategic traditional land use practices, Indigenous Cultural reference states seek conservation strategies that manage fire, invasive species and biodiversity loss (e.g. Ban et al., 2020; Ens et al., 2015; Molnár et al., 2020).

These reference states, which include the pre‐Columbian reference applied in the Americans and European countries, or pre‐European applied in Oceania and Canada, mark disturbances related to introducing non‐native or alien species, especially plant and animal species used as food sources (Kühn et al., 2004; Pyšek et al., 2004) and altered landscape configuration and composition as a direct result of acclimation and agricultural practices. These reference states have been used to report on the extent of vegetation change in Australia (e.g. Bradshaw, 2012; Gibbons et al., 2009; Mendel, 2002), Africa (e.g. Aleman et al., 2018; Cowling et al., 2003) and North America (e.g. Au et al., 2020; Keddy & Drummond, 1996; McEwan et al., 2011; Motzkin & Foster, 2002). There are many examples where former climates, landscapes and vegetation have been extrapolated from dendrochronology, palynology, peat, sediment or ice cores. Some clear evidence of pre‐Columbian and pre‐European reference states can be derived from historical survey records, tree stumps or pattern in vegetation clearing for agriculture (e.g. Aleman et al., 2018; Bjorkman & Vellend, 2010; Fensham & Fairfax, 1997; Jackson, 2013; Lunt, 2002; Seabrook et al., 2006; Silcock et al., 2013; White & Mladenoff, 1994). The Indigenous Cultural reference states are tractable because there are often clear connections between altered disturbance patterns induced by non‐Indigenous colonization and the decline in ‘naturalness’ and an increase in extinction rates and invasion by non‐native species.

### 2.3 Pre‐Intensification reference states

The Pre‐Intensification reference state represents critical temporal break‐points prior to amplified rates of ecological change attributed to human disturbance (Heller & Hobbs, 2014; Lewis & Maslin, 2015). For example, industrialization and extraction of fossil fuels have triggered global transformative changes (Steffen et al., 2011). However, this reference state is not confined to impacts resulting from the Industrial Revolution. Evidence of the long history of human use of metals and subsequent metal pollution such as copper smelting used during Roman Empire (circa 2,000 years before present) can be detected in ice cores (Lewis & Maslin, 2015). Although intensification initially commenced in localized areas, impacts of human modification and disturbance can be detected using local and global environmental indicators.

In contrast to the Pre‐Human and Indigenous Cultural reference states, baselines for Pre‐Intensification reference states can be clearly defined using environmental markers at global, regional and local scales. For example, spikes in radionucleotides and atmospheric ^14^C determine the onset of global intensification (Lewis & Maslin, 2015; Turney et al., 2018). Measuring environmental markers, such as nitrogen and phosphorous from fertilizers (Lu & Tian, 2014) or heavy metals (Shotbolt et al., 2007), offers a tractable approach to identifying Pre‐Intensification reference states. For example, the European Water Framework Directive criteria define ‘*concentrations of specific synthetic pollutants should be close to zero*’ as a concrete descriptor of Pre‐Intensification reference states, and these markers are easier to measure compared with other criteria, such as biotic integrity (Hering et al., 2010).

### 2.4 Hybrid‐Historical reference states

The Hybrid‐Historical reference state represents a reconstruction of past conditions based on current patterns. This reference state moves towards bridging historical and contemporary theoretical perspectives (Figure 1) and perceives historical states through a contemporary lens. Hybrid‐Historical is synonymous with ‘near‐natural’, ‘long‐undisturbed’, ‘unmodified’, ‘relatively undisturbed’ or ‘least‐disturbed’. Underpinning the Hybrid‐Historical reference state is an assumption that least disturbed ecosystems retain a majority of native biota (Landres et al., 1999); are most resilient to disturbance (Holling, 1973; Naeem & Li, 1997; Yachi & Loreau, 1999) and thus support better conservation outcomes. Unlike Pre‐Human, Indigenous Cultural and Pre‐Industrial, the Hybrid‐Historical reference state is not defined by time stamps and may combine evidence from multiple reference states shown in Figure 1. For example, Hybrid‐Historical reference states can be described from on‐ground, extant evidence in conjunction with evidence of post‐intensification anthropogenic disturbance, such as vegetation clearing, timber harvesting, the extent of change in the composition of vegetation, cultivation, fertilizer application or livestock grazing. Tools like historical aerial photography can be used to trace patterns of change. Often this reference state is identified by combining both current and reconstructed historical patterns of disturbance to identify least‐disturbed and long‐undisturbed reference states (e.g. Gibbons et al., 2008; Hessburg et al., 1999; Seddon et al., 2011). An alternative approach to measuring on‐ground evidence of disturbance history has been the use of land tenure (such as protected areas, nature reserves and national parks) as a proxy (e.g. Scholes & Biggs, 2005; Sinclair et al., 2002). Landscape condition assessments have used national conservation reserves (e.g. Harwood et al., 2016) or identified areas based on their distance from human populations or infrastructure (e.g. Allan et al., 2019; Watson et al., 2018) as the benchmark for identifying least disturbed. However, protected areas are not always minimally disturbed and are often not representative of a majority of ecosystems (Joppa & Pfaff, 2009). Some areas with the greatest biodiversity value are in unprotected tenures (Archibald et al., 2020; Clements et al., 2019; De Vos & Cumming, 2019) and face some of the greatest threats (Myers et al., 2000).

Hybrid‐Historical reference states can be differentiated from Indigenous Cultural reference states in that they do not explicitly account for prior Indigenous ecological management practices and how this may have influenced biotic relationships with climate and landscape. The Hybrid‐Historical reference state is appealing because it can overcome some of the uncertain or unquantifiable characteristics of historical ecosystems (Balaguer et al., 2014).

## 3 LIMITATIONS OF HISTORICAL REFERENCE STATES

Historical perspectives of ecosystems gleaned from multiple lines of evidence have been essential for developing hypotheses for how ecosystems evolve and function (Swetnam et al., 1999). Historical drivers of change have been vital in navigating how ecological processes determine contemporary patterns of biodiversity and predicting changes in ecosystems. However, historical reference states do not explicitly target the goal of maximizing biodiversity conservation outcomes in contemporary ecosystems; at best they hypothesize that attaining a historical reference state will have a co‐benefit of maximizing biodiversity. Within the specific context of setting targets for maximizing biodiversity through ecosystem management, conservation and restoration actions, we argue that there are three key limitations to the use of historical reference states in contemporary ecosystem management. First, historical reference states are often unmeasurable. Second, they are almost always unfalsifiable. Finally, historical reference states may be unattainable in contemporary landscapes. While this final point is not important in the context of describing change over time, it is a significant limitation in the context of setting targets for maximizing biodiversity outcomes through ecosystem management, conservation and restoration actions.

### 3.1 Historical reference states are often unmeasurable

One of the key criticisms of historical reference states is that they are difficult to quantify. Prior ecological patterns and their associated climate, disturbance regimes and biological interactions may not be represented in present‐day analogues. Therefore, historical reference states must be indirectly inferred, extrapolated or reconstructed, often from fragmented evidence. Long‐term, detailed, continuous data are lacking for most species, communities, ecosystems and few population trends have been studied for more than 100 years (Bonebrake et al., 2010; Mihoub et al., 2017; Vihervaara et al., 2013). Detailed, site‐specific biological records rarely pre‐date human disturbances (Balaguer et al., 2014; Hobbs et al., 2014) and where long‐term records have been collected, rarely do they adequately describe detailed community structure and species composition. As a result, reconstructions of species' assemblages or ecosystems may be too imprecise, incomplete or inadequate to inform ecosystem‐level decisions (Stoddard et al., 2006) and are likely to be biased given the non‐random nature of human disturbance.

Because historical reference states and their benchmarks are often pieced together from sparse information, they often rely on expert or best professional judgement (Borja et al., 2012; Oliver et al., 2007; Weisberg et al., 2008). However, these opinions can be uncertain, value‐laden, subjective and prone to counter‐perspective and discrepancy (Burgman et al., 2011). The bias, validity and reliability of expert judgement are especially problematic when it is difficult to assess the transparency and repeatability of the methods (Drescher & Edwards, 2019). Consequently, reproducing benchmarks generated by expert opinion may lead to discrepant results, wide variations in estimates or contested debate.

In some contexts, time‐stamps can also be challenging to quantify or confirm. Pre‐Human and the subsequent Indigenous Cultural reference states are difficult to pinpoint with certainty because there have been multiple waves of both Indigenous and non‐Indigenous colonization (Dubois et al., 2018) and these patterns have not been uniform across whole landmasses. Locally and regionally, reference states would have varied time‐stamps owing to the radiation of human migrations, and therefore a specific time‐stamp is ill‐defined and difficult to discern. As an example, Dutch and Spanish and Macassan people arrived on the Australian continent as early as 1606, well before 1788 British colonization. The subsequent patterns of British colonization differed over all parts of the continent. In contrast, Pre‐Intensification reference states can often be time‐stamped consistently and accurately using environmental markers and can be correlated with ecological or biotic patterns relevant to biodiversity conservation and management (Zhang et al., 2018).

The Hybrid‐Historical reference state neither depends on explicit time‐stamps nor estimates of historical structure and composition of biota. However, it does rely on consistently defining ecosystems that are least disturbed and quantifying disturbance history, which can be challenging and a key source of uncertainty (Nagel et al., 2007). Hybrid‐Historical reference states also rely on space‐for‐time as a substitute for temporal data (Cava et al., 2018; Lotze & Worm, 2009; Symstad & Jonas, 2014). Space‐for‐time experimental designs are common but criticized because they assume ecological observations are at equilibrium (Damgaard, 2019). However, this assumption relies on knowledge of temporal dynamics which are difficult to estimate in the absence of long‐term data (De Palma et al., 2018). Space‐for‐time substitutions are difficult because they require large unaltered areas or intact regions that represent the range of conditions in the contemporary ecosystem. However, large, intact, remote and protected ecosystems are often temporally and spatially biased (Damgaard, 2019; Joppa & Pfaff, 2009; Negret et al., 2020) and do not account for non‐random human disturbance.

### 3.2 Historical reference states are often unfalsifiable

Because historical reference states are often unmeasurable, they generally cannot be tested, evaluated and improved by falsifiable enquiry (McCarthy et al., 2004). This is a critical shortfall of most historical reference states because the foundation of transparent and rigorous science is repeatable methods and measurements (Wolman, 2006). In the absence of a systematic and comprehensive set of empirical samples to describe historical reference states, it is problematic to prove any meaningful relationships between biodiversity values and reference states. We highlight that Pre‐Human reference states are the least likely to be falsified and the Hybrid‐Historical reference state approaches the premise of falsifiability. In Hybrid‐Historical reference states, ecosystems can be identified as ‘undisturbed’ and hypotheses can be tested to ascertain if higher biodiversity values are observed within these Hybrid‐Historical reference states relative to other locations within the same ecosystem.

### 3.3 Historical reference states may be inappropriate because they are unattainable

Most of the Earth's ecosystems have been adversely affected by anthropogenic pressures (Butchart et al., 2010). When historical reference states are perceived through a lens of either ecological or social values or both, then returning to the former historical state may sometimes be unattainable. For example, Zweig and Kitchens (2010) argue that aiming to restore the Florida Everglades to a pre‐1880 historical state, prior to extensive changes in the hydrology, vegetation and soils, would adversely impact on current livelihoods, food security, economies and societal conditions. In instances such as this, proliferating population and intensifying urban and agricultural development cannot be reconciled with the restoration of historical states. The ecological–societal nexus conflicts with the potential to ‘return’ to a historical reference state.

Current biological and abiotic pressures can also hinder passive recovery or make efforts to pursue active restoration or re‐introductions untenable. Where the global biotic and abiotic conditions (such as climate, changes in flood regimes, changed fire intensity and frequency, warming ocean temperature, rising sea level, increased atmospheric CO_2_ or overexploitation) cannot be reinstated, or are poorly known, historical reference states may be unattainable and unrepresentative of contemporary habitats or biodiversity (Corlett, 2016; Rohwer & Marris, 2016). In some instances, local abiotic conditions have moved beyond remediation (e.g. altered soil properties, nutrients, pH) or biotic assemblages (species redistribution, habitat alteration and loss, increase in invasive species, loss of ecosystem‐engineering species or extinct mega‐fauna) cannot be reinstated. For example, as a result of rising sea temperatures, Hughes et al. (2017) consider the recent historical conceptual frame as a non‐viable option for managing contemporary coral reefs. Similarly, Bond and Midgley (2012) postulate that historical fire regimes may be inadequate for maintaining tree‐grass dynamics in humid savannah ecosystems as a result of the near doubling of atmospheric CO_2_.

Hobbs et al. (2014) argue that many ecosystems have so radically changed from any recognizable historical reference state that conservation outcomes in contemporary ecosystems need an alternative approach for setting conservation management and restoration goals. By classifying ecosystems based on irreversibility, Hobbs et al. (2014) provide a framework for evaluating where and if historical reference states are attainable.

Where societal, abiotic and biological constraints can be reversed, ecosystems could be managed as hybrid ecosystems based on historical reference states. Whereas ecosystems that arise as a result of irreversible barriers may need to be managed as novel ecosystems with no clear historical analogue. This approach is a useful guide to construct a decision framework as it outlines how management goals can be evaluated. Where the conservation, management and restoration goal is to maximize biodiversity conservation and restoration outcomes for contemporary ecosystems, consideration and decoupling of the societal and biological constraints will help determine feasible and appropriate applications and inform how, where and if historical reference states are attainable.

## 4 AN ALTERNATIVE THEORETICAL PERSPECTIVE: CONTEMPORARY REFERENCE STATES

To overcome the complications and limitations associated with defining and measuring historical reference states, and with maximizing biodiversity conservation and restoration outcomes for contemporary ecosystems, we promote an alternative theoretical perspective based on the concept of ‘contemporary reference state’ (Figure 1). A transition away from historical disturbance‐driven reference states to contemporary biodiversity value‐based reference states signals an opportunity to assess diversity‐driven characteristics of contemporary ecosystems and overcome the complications and limitations of the disturbance‐driven approach. Kopf et al. (2015) also recognize the benefits of contemporary reference states and propose Anthropocene baselines as an alternative framework for contemporary ecosystems. However, unlike Kopf et al. (2015), we propose a shift from a disturbance‐driven philosophy to a diversity‐driven philosophy.

Our conceptual framework for contemporary reference states extends the ‘Best‐of‐What's‐Left’ concept (Stoddard et al., 2006) in that it does not contemplate historical reference states, but orientates to known, extant states that are identified by high levels of current biodiversity values within and among contemporary ecosystems. The concept centres on a ‘more diversity is better’ philosophy and builds on the ‘Best‐on‐Offer’ reference state relative to other locations within the same ecosystem as proposed by Eyre et al. (2006) whereby biodiversity values that exist under contemporary conditions can be clearly defined and represent an approach to quantifying the status of biodiversity.

Given no community consists of the same species in equal abundance, we argue the contemporary reference state concept should be based on a standardized assessment of native species diversity and measured relative to the native species diversity within the same ecosystem with consistent climatic, edaphic, topographic and biogeographic characteristics (Sinclair et al., 2002; Symstad & Jonas, 2014). By assessing native species diversity using standardized approaches and relative to a comparable community or ecosystem, the contemporary reference state approach can account for the inherent turnover in biodiversity that occurs between spatial scales, communities and ecosystems. That is, contemporary reference states and the benchmarks that define them are specific to ecological communities assessed at consistent spatial scales. Box 2 illustrates one recent attempt to identify and operationalize the contemporary Best‐on‐Offer reference state. In this operational example, sites with higher numbers of native plant species and greater structural complexity (relative to other sites of the same vegetation type) were used to identify the Best‐on‐Offer reference state as an approach to improving biodiversity conservation outcomes within south‐eastern Australia (Yen et al., 2019).

The ecological justification for describing a Best‐on‐Offer reference state can be supported by theoretical underpinnings which hypothesize that a site with higher levels of native species alpha‐diversity has increased stability over time (Hector et al., 2010; Loreau et al., 2001; McCann, 2000; Tilman et al., 2001, 2006); stability over space (Hector et al., 2010; McCann, 2000) and adaptive capacity (Baho et al., 2017). Sites with greater alpha‐diversity have greater ecological integrity (Brooks et al., 1998; Oliver et al., 2014); higher productivity (Tilman et al., 2012) and provide a greater diversity of ecosystem services (Hooper et al., 2005) and functions (Schwartz et al., 2000). The ‘more diversity is better’ paradigm moves towards an insurance policy approach and assumes where ecosystems are replete they are more resilient (Holling, 1973; Naeem & Li, 1997; Yachi & Loreau, 1999). However, by defining the contemporary reference state at the biome, ecoregion or ecosystem level, the concept also accounts for beta‐ and gamma‐diversity (Whittaker, 1967). We emphasize here that diversity of native species is a key consideration, not overall species diversity.

## 5 BENEFITS OF DIVERSITY‐DRIVEN CONTEMPORARY REFERENCE STATES

A diversity‐driven contemporary reference state approach has a number of strengths. It does not need to make assumptions about disturbance history, natural or historical range of variability, nor the interactions between human‐induced and natural disturbances. Importantly, diversity measures are comparative within the same ecological units which can account for describing reference states in different conditions, including naturally depauperate ecosystems. Contemporary reference states can be specifically defined across time and space (Balaguer et al., 2014).

In addition, the contemporary reference state concept can potentially overcome some of the shortfalls we have outlined with the historical reference state concept because the diversity‐driven approach allows us to quantify biodiversity values using empirical data collected from within existing ecosystems. It does not require expert opinion to estimate what is ‘good’ quality or ‘higher’ integrity or ‘maximum’ biodiversity value. It circumvents the problems with using expert opinion, in which evaluation of benchmarks is based on subjective counter‐opinion (Sinclair et al., 2015). The empirical description of reference states enables a more transparent, rigorous, repeatable and falsifiable approach to assessing conservation and restoration targets (Box 1) and moves us away from a value‐laden approach to evaluating ecosystems (Wolman, 2006). This is important when removing the subjectivity comparing different biodiversity outcomes that may be competing or contentious.

Several additional benefits of shifting to a Best‐on‐Offer reference state include the ability to locate reference sites spatially and temporally. Reference sites can be identified from underlying data and can conceivably become the ‘exemplar’ that defines and represents the on‐ground characteristics of the Best‐on‐Offer reference state. Exemplar sites can be measured and monitored to gain a better understanding of how biodiversity composition, structure and function at reference sites change over time (Hiers et al., 2012). This could include year‐to‐year variation as well as longer‐term trends that might be associated with large‐scale shifts in climate, biotic interactions or disturbance regimes. Furthermore, benchmarks can be refined by ongoing assessment and monitoring of exemplar sites which has benefits of delivering the potential for dynamic benchmarks that represent seasonal and climatic changes (Yen et al., 2019). The timeframe of the reference state baseline is clearly articulated because this approach is based on the underlying data. Therefore, benchmarks to define the contemporary reference states can develop and improve as new data become available. Moreover, the contemporary reference state concept is scalable, and where data are available, this approach can be applied at local, regional and global scales providing that reference states are defined relative to individual ecological units.

BOX 2 **Measuring diversity‐driven contemporary reference states**

**An operational approach for defining contemporary Best‐on‐Offer reference states to support the Biodiversity Conservation Act in New South Wales, Australia**

Foliage cover and species richness of native plants provides information on vegetation structure and composition and are frequently used as surrogates for biodiversity (Westgate, Tulloch, Barton, Pierson, & Lindenmayer, 2017). With the ever‐expanding repositories of floristic data that contain inventories of plant species, there are opportunities to develop empirical benchmarks using existing vegetation data. Here, we demonstrate how floristic data were used to define the Best‐on‐Offer benchmarks to support and implement the Biodiversity Assessment Method under the Biodiversity Conservation Act 2016 (Yen et al., 2019). Under the New South Wales Biodiversity Conservation Act 2016, Vegetation Integrity was identified as a key biodiversity value and defined according to benchmarks for foliage cover and richness of native plant growth forms along with other habitat attributes.

**Screen and stratify**

**FIGURE 2:**
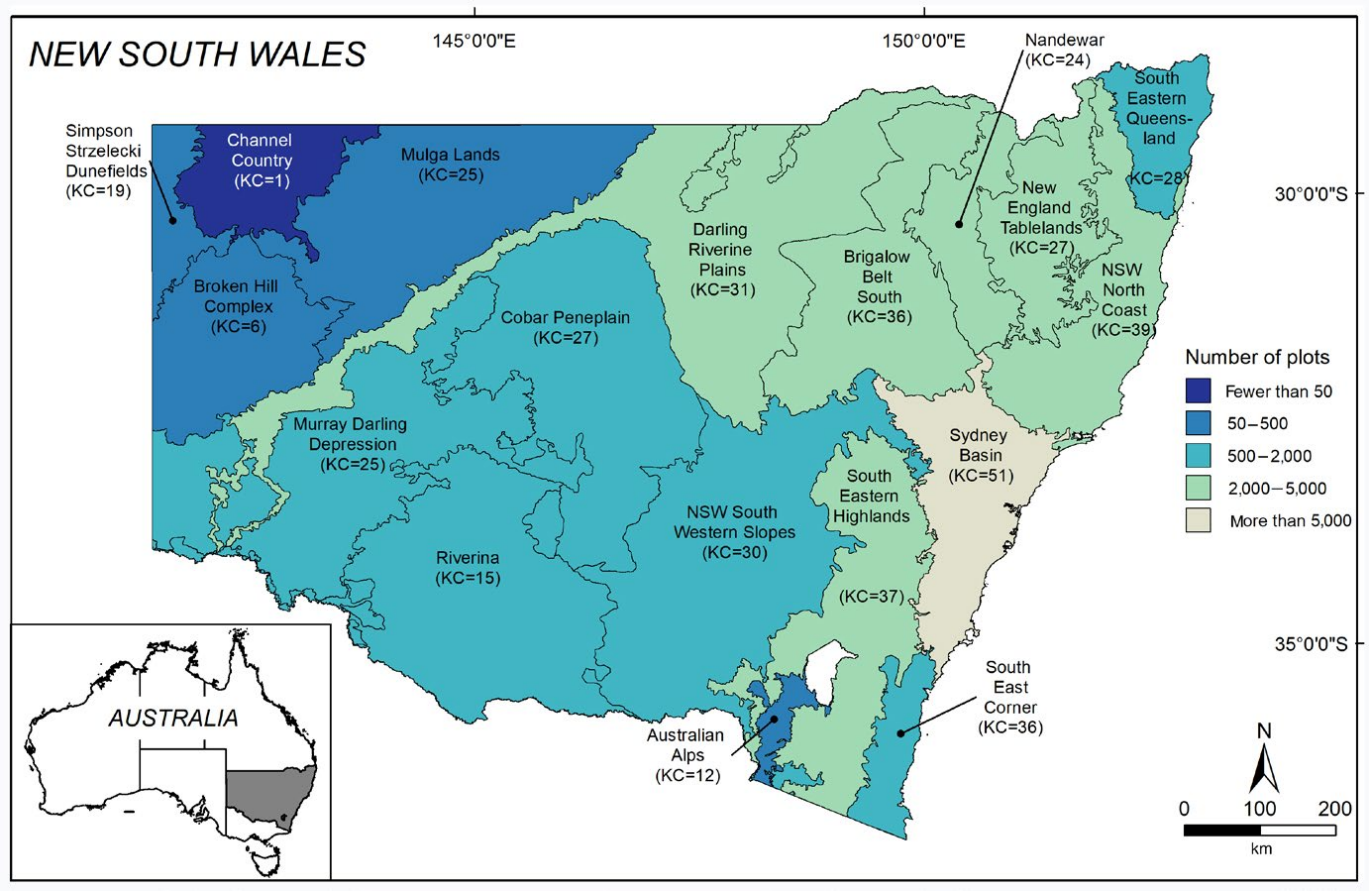
Eighteen broad geomorphological units within the study area. Numbers shown in parentheses are the number vegetation communities (KC) with floristic plot data

From the BioNet Atlas (www.bionet.nsw.gov.au) we extracted approximately 70,000 archived floristic plots. We screened these data and selected only plots that were comprehensibly and systematically surveyed within a fixed 0.04 ha area (approximately 36,000 plots) and excluded sites surveyed prior to 1970. We classified plots by vegetation communities (Keith, 2004). We then we used spatial analysis to stratify the study area by geomorphological units (Figure 2). The combination of vegetation community and geomorphological unit was used to create regional vegetation classes (RVCs).

**Synthesize and operationalize**

For each of the approximately 36,000 plots we allocated all native vascular plant species to one of six growth forms, trees, shrubs, grasses, forbs, ferns and others (Oliver et al., 2019), and calculated the structural (summed foliage cover of species within each growth form) and compositional (species richness within each growth form) each plot. We used modelled upper quantiles of the data distributions to estimate contemporary, Best‐on‐Offer benchmarks for growth form cover and richness (Yen et al., 2019). Importantly, benchmarks are specific to the structure and composition of growth forms and vary across the landscape according to the RVC (combined geomorphology and vegetation community). Further information is provided at https://www.environment.nsw.gov.au/topics/animals‐and‐plants/native‐vegetation/vegetation‐condition‐benchmarks.

This case study demonstrates how plot level data can be used to describe a contemporary Best‐on‐Offer reference state to maximize biodiversity conservation and restoration outcomes in existing ecosystems and could be extended, using alternative data sources to account for beta‐ or gamma‐diversity between spatial scales, communities and ecosystems.

## 6 LIMITATIONS OF THE DIVERSITY‐DRIVEN CONTEMPORARY REFERENCE STATE APPROACH

### 6.1 Describing patterns, not processes

One of the most challenging aspects of defining a contemporary reference state is to understand the complex nature of drivers of change. Critically, a contemporary benchmark paradigm does not suggest ecologists should ignore prior patterns and processes. Central to this endeavour is an improved understanding of long‐term dynamics of ecosystems shaped by slow (such as climate change) and fast (such as rapid socioeconomic development) drivers over various timescales. Without an understanding of both past and present ecological processes, contemporary reference states merely describe observed patterns. That is, we acknowledge that past processes play an essential role in driving contemporary patterns of diversity. Therefore, the local conditions in which contemporary reference sites occur, and past disturbances and management to which these sites have been exposed, provide insight into the conditions under which the contemporary reference state may be attained. As outlined above, the contemporary reference state might still be unachievable at some locations because underlying processes cannot be reinstated, or are outside of human influence.

Another perceived shortfall of a contemporary reference state is accepting that the best representation of some ecosystems is altered or degraded, and not representative of an ‘ideal’ state, and therefore a contemporary conceptual frame lowers or compromises outcomes for biodiversity (Soga & Gaston, 2018). We acknowledge these concerns (sensu shifting baseline syndrome Pauly, 1995) and argue that the application of contemporary benchmarks requires their explicit consideration. As shown in Box 2, one approach to confirming change in baselines would be to initiate a network of contemporary reference sites to measure trends in diversity. Villnäs and Norkko (2011) argue that shifting baselines are an inevitable consequence for all ecosystems across time and space, and suggest that data‐driven baselines may offer a transparent solution. Given that change is unavoidable, yet the rates of change are difficult to quantify with certitude, we anticipate that reference states for novel and no‐analogue systems will require careful attention (Hobbs et al., 2009, 2014). Data‐driven reference states pave an approach to debate or confirm how future states are assessed.

A further consideration when defining reference states is the spatial scale and extent of the ecosystem. Where ecosystems are broad and data are sparse, the confidence in setting empirical benchmarks will be lower and this is particularly important when reference states and benchmark concepts underpin operational biodiversity assessments. Furthermore, the types of data available, their temporal span, the variability within and between different ecosystems and geographical locations are important considerations when building diversity‐driven reference states. For example, ephemeral, annual, sparse, rare, migratory or mobile species will be more difficult data types to derive diversity‐driven benchmarks.

## 7 CONCLUSIONS

Here we synthesize a conceptual framework and provide a ‘road map of reference states’ to clarify an approach to define and assess the current status and better manage the expected trends in the decline of biodiversity and degradation of habitats. When choosing ‘which state’ or ‘which baseline’, we show that different reference states are used in different contexts. Distant historical reference states mark a baseline to assess former presence and abundance of species, identify extinctions, reconstruct former habitats or reinstate past disturbance regimes. Where reference states use more current information and are centred on less distant timeframes, more refined knowledge on structure, composition and function can be generalized. Yet, even when more sophisticated information is available, historical reference states may not best address the need to maximize biodiversity outcomes or inform restoration goals in contemporary ecosystems. The contemporary reference state framework is a major conceptual shift that focuses on current ecological patterns and prioritizes conservation of areas with higher biodiversity values. In contrast to historical reference states, contemporary references states are measurable and falsifiable and can be tested to assess if they do represent high values of biodiversity.

Human‐dominated ecosystems have undergone rapid and extensive transformations in the past 50 years and are forecast to accelerate over the next 50 years (Millennium Ecosystem Assessment, 2005). We foresee contemporary reference states as an operational tool for policy‐makers and practitioners needing to assess biodiversity values consistent with maximizing biodiversity conservation and restoration outcomes in contemporary ecosystems.
